# Scaling Down the PGCM Scale to Assess Views on Ageing More Efficiently: Finding a New Alternative

**DOI:** 10.3390/ijerph17249414

**Published:** 2020-12-15

**Authors:** Tomáš Doseděl, Tereza Menšíková, Lucie Vidovićová

**Affiliations:** Faculty of Social Studies, Masaryk University, Joštova 10, 602 00 Brno, Czech Republic; 427682@mail.muni.cz (T.M.); vidovicova@fss.muni.cz (L.V.)

**Keywords:** views on ageing, measurement, Philadelphia Geriatric Center Morale Scale, PGCM, ATOA, morale, quality of life, positive outlook, POLA

## Abstract

The aim of the study is to present a new and efficient way of measuring the quality of life among older populations, with special attention to morale, positive outlook on life and ageing. The measure is based on the Philadelphia Geriatric Center Morale Scale (PGCM), originally consisting of 22 items. The growing numbers and proportions of older people among European populations has increased the need to obtain more reliable data on their needs, values, life experiences and overall quality of life. Using data from six surveys conducted in the Czech Republic, we have formulated a three-item positive outlook on life and ageing (POLA) scale. Our analyses are divided into three steps: (1) constructing the scale and testing its internal consistency; (2) testing the scale’s external validity using mean comparisons and correlation coefficients; and (3) determining the factors affecting a positive outlook on later life, such as gender and education. We have confirmed that the three-item approach to measuring positive outlook on ageing as a part of morale is highly applicable to surveys, producing compelling results in assessing important quality-of-life sub-concepts, such as subjective health, subjective age, and loneliness.

## 1. Introduction

The demographic change resulting in growing numbers and proportions of older people in European populations [1] has increased the need for better understanding the key aspects of active and successful ageing [2,3], life satisfaction, and quality of life among older adults. Even though population ageing does not necessary imply a declining living standard, a decrease in economic productivity and increased costs of social and health care [4], the systemic consideration of public policies reflecting the well-being of older adults is warranted across countries [4,5,6] in order to address the various challenges related to the multifaceted and multi-layered changes brought about by ageing processes for both societies and individuals.

Reflecting upon the well-being and quality of life among older populations using psychological measures represents a long-term interest within the field of gerontological and geriatric research [7,8,9,10,11,12,13,14]. Emphasis on the subjective experience of individuals and their psychological well-being has become the basis for morale and quality-of-life studies [15]. The most popular measure among gerontological studies, alongside the Life Satisfaction Index and the Affect Balance Scale, is the Philadelphia Geriatric Center Morale Scale (PGCM/PGCMS) [16]. As confirmed recently by Klusmann et al. [7], the PGCM scale is the second most often cited measure for assessing views on ageing, being quoted more than 783 times in the Web of Science database, with an average of 18 citations per year.

In this study, we seek an alternative way of measuring the quality of life among older populations, with special attention to morale. We propose a new succinct measure of positive outlook on life and ageing, reflecting aspects of the greater socio-gerontological concepts of happiness, optimism, energy, and sense of purpose. The original PGCM scale consists of twenty-two items, creating considerable demands in terms of space, time, and expense when used in surveys among older people. Therefore, many variants have been proposed with fewer factors [17] or items, including, for example, seventeen- [18], fifteen- [19], or eleven-item variants [20]. Several studies have also used just one subscale of the PGCM, the five-item Attitude Towards Own Ageing (ATOA), as a stand-alone measure to study the self-perception of ageing as an important indicator of both subjective and more objective later life conditions and outcomes [21,22,23,24,25].

Using data from six different surveys conducted in the Czech Republic, we formulate a three-item morale scale based on the PGCM and the ATOA as a departure point, to propose a succinct yet useful indicator for measuring positive outlook on the process of ageing and later life. We proceed from the premise that a positive response to a zest for life enables people to adapt more positively to changes in life and to accept future events in more accommodative ways; previous studies have suggested that if people cope with old age more successfully and approach life with greater optimism, they also feel more useful and show higher levels of life satisfaction [26,27]. The phenomenon that we might call “an awareness of ageing processes” has been studied from various angles. Empirical research identifies several antecedents of awareness of ageing processes, such as sociodemographic “background” variables, physical health and physical functioning, cognition, psychological well-being and mental health, psychological variables (e.g., personality, anxiety), and life events: “In general, more positive manifestations on these variables are accompanied by a more positive perception and evaluation of the ageing process. Moreover, awareness of ageing processes is longitudinally linked to important developmental outcomes, such as health, cognition, subjective well-being, and mortality” [28].

For this particular article, we have tested the soundness of our composite indicator of positive outlook on later life in different thematic contexts. We take particular interest in gender and other socioeconomic determinants, as well as subjective age [29] and loneliness, as crucial indicators of quality of life [30]. Our analyses are divided into three steps: (1) constructing the positive outlook on later life and ageing (POLA) scale and testing its internal consistency, (2) testing the scale’s external validity using mean comparisons and correlation coefficients, and (3) determining the factors related to a positive outlook on ageing. This study suggests that the three-item approach to measuring morale by POLA is applicable in surveys, and moreover, by lowering the burden placed on older respondents, it has the potential to produce better survey results among older populations.

### Philadelphia Geriatric Center Morale Scale—The Inspiration

The original PGCM scale developed by Lawton [31] viewed subjective well-being as a multidimensional concept and comprised 22 items measuring six separate dimensions of morale. The need for a multidimensional approach to quality of life, along with an accessible format and wording suitable for the senior population, were the main reasons for establishing a new type of measurement [32]. In his later publication, Lawton, referring to the work of Morris and Sherwood [18], revised the PGCM scale and presented a three-factor structure of 17 items representing the dimensions of morale: Attitude Toward Own Aging, Agitation, and Lonely Dissatisfaction [32]. A version of the PGCM scale in British English [33] was recommended for its high internal consistency and reliability by the United Kingdom’s Royal College of Physicians and the British Geriatric Society in 1992 as an assessment scale focused on the morale dimension of the quality of life among older populations [34]. The high correlation scores with the Life Satisfaction Index Z [35], the Short-Form Health Survey [15], the Bradburn Affect Balance Scale, and the Life Satisfaction Index [16,18,36] led to the PGCM scale becoming a widely used tool for the measurement of subjective well-being [37].

The PGCM scale comprised of 17 items later became the most used variant of the scale for measuring the quality of life among older populations [33,38,39,40,41,42,43]. The various applications over the years across research fields and countries such as Sweden [39,40,41], Spain [44], Finland [38,40], Hong Kong [17], Japan [19,45,46], Israel [47], Malaysia [48], and Turkey [15] eventually led to modifications of the scale. These modifications aimed to improve several of the scale’s aspects, to consider possible measurement errors, and to adjust the questionnaire to fit different cultural backgrounds. However, the differences among applications vary to such an extent that full comparison is very complicated.

The original formal composition of the questions was modified, and some items were rephrased during the transition from the Lawton version to the British English and Swedish versions [41]. Changes were also made to the ATOA five-item subscale, which comprises dimensions such as perceived usefulness, happiness, pep, and positive/negative feelings toward the past. An interesting comparison was found by Levy, Slade, and Kasl [21], when their factor analysis showed that the ATOA subscale loaded highly (above 0.8) on a single factor. The same result emerged from the studies by Liang and Bollen [19] and by Kim, Jang, and Chiriboga [23]. Jung and Siedlecki [49] obtained good results with a four-item subscale. However, a recent review of measurement properties [50] has pointed out that the numerous modifications and inconsistencies of the ATOA have made an assessment of the quality of the subscale very difficult.

The authors of the review [50] also raise concerns about the use of the ATOA and do not recommend subscale as a measure of ageing self-stereotype. On the other hand, according to recent studies, the ATOA, as an example of an indicator of awareness of ageing processes, produces compelling results on various aspects of the lives of older adults [27,28]. The quest to find better and more usable indicators for morale and positive outlook on ageing and later life is, therefore, still an open challenge without a clear consensus. We therefore take this opportunity to propose a new brief scale inspired by the PGCM’s ATOA, which we have utilized in several surveys on the various aspects of older people’s lives and present it here for further discussion with the experts in the field.

## 2. Materials and Methods

In order to examine the options for alternative measures of positive outlook on later life as a possible indicator of positive ageing, we used data from six different surveys. Each survey was conducted in the Czech Republic as a part of separate empirical project, carried out between 2007 and 2016. The data collection was commissioned to professional social research and market research agencies observing ESOMAR standards, respecting the ethical standards for quantitative surveys, and ensuring that informed consent was obtained prior to the interviews. The surveys were conducted as parts of different research projects at the Faculty of Social Studies of Masaryk University or the Research Institute for Labor and Social Affairs, sharing the same principal investigator and core scientific group (for more details on the original research projects, please see https://starnuti.fss.muni.cz/). Data for all surveys were collected using the PAPI method and respective interviewees were selected within the respondent networks maintained by the agencies. The interviewees were exclusive for each of the data collection events, and one survey collected 290 variables on average (minimum 255 and maximum 315), with interviews lasting approximately 60 min on average.

The Ageism 2007 survey analysed the perception and prevalence of age discrimination in different areas of social life among the adult population. Information on attitudes, values and experiences related to concepts, such as age salience, perceived and experienced age discrimination, and attitudes towards ageing and older people were collected. Here, the POLA scale as an ad hoc construct derived from the PGCM was first used to test the hypothesis that people with a more positive outlook on later life have also “better” scores in different measures of the awareness of aging processes, including subjective age. Five years later, the survey was administered, under the name Ageism 2012, on a different set of respondents. The Role Overload Survey in 2014 interviewed people aged 50–70 years about their prevalent, conflicting, and rewarding social roles, with special attention to the grandparental role. In 2011, a survey of older people living in large cities (Quality of Life in the City—QinCity) investigated housing conditions, mobility, relations with neighbors, social and instrumental activities, perceptions of changes to physical space and its age-friendliness, and other relevant enviro-gerontological issues related to place-based quality of life. The survey had two parts—QinCity 1 was conducted in the 14 largest cities in the Czech Republic, while QinCity 2 focused only on Prague (the Capital), Brno, and Ostrava to strengthen the dataset [51]. Very similar topics were explored in the older populations of small towns and rural areas in the RURAL Survey (2016).

For the purpose of this article, the data from all of the abovementioned surveys were combined into one data file in order to gain a well-populated sample and test the soundness of the new proposed measure in different thematic contexts. Only the relevant variables were brought to the new study file and altered as follows. All of the variables used were transformed to make them comparable (for example, if the codes for men and women were different in particular surveys, they were recoded to the same values across all surveys). Data from respondents younger than 60 years were deleted from the resulting file and therefore omitted from all analyses. The resulting dataset had 5190 respondents, 43 per cent men and 57 per cent women. The majority of respondents (41 per cent) had received vocational training, a quarter (25 per cent) had completed full secondary education with the leaving exam (“maturita”), and about one-tenth (11 per cent) had university degrees. The basic characteristics of the original and resulting datasets are summarized in Table 1.

We utilized a three-fold analytical strategy. First, a three-item positive outlook on later life and ageing (POLA) scale was constructed, and its internal consistency was tested using Cronbach’s alpha test. (If the alpha is acceptable, the constructed scale measures a one-dimensional concept.) Second, external validity tests were performed by showing the relation between the three-item POLA scale and selected variables. If the constructed variable truly measured the “quality” of the outlook on ageing, it should differentiate the respondents according to their physical age, sex, education, subjective measure of loneliness as a proxy for social isolation, and other selected characteristics. Lastly, the determinants of the three-item POLA scale were estimated using linear regression modelling.

## 3. Results

As the main dependent variable, we constructed a three-item positive outlook on ageing scale. This scale is inspired by the Philadelphia Geriatric Centre Morale Scale [32] and the ATOA, in which morale is a multidimensional concept defined as future-oriented optimism or pessimism regarding the problems and opportunities associated with ageing [41]. Our measure tested here consists of a positive outlook on the subject’s ageing, feelings of usefulness, and feelings of happiness, including a reflection of the comparative dynamism of the life-course experiences of the individual respondent.

The scale is computed as the arithmetic mean of the three items—answers to three questions concerning respondents’ feelings: “Do you have more or less pep for life than a year ago?”, “Do you feel less or more useful than you felt when you were younger?”, and “Are you more or less happy as you get older?”. These items were inspired by the subscales of the PGCM and the ATOA and were tested by [32] as good predictors of expected outcomes. All answers were coded as 1—“things are worse”, 2—“things are the same”, and 3—“things are better” than before. The introduction of a middle variant has brought forward an aspect of the development dynamic, in comparison to the ATOA’s “yes/no” answer options. Therefore, the resulting mean varies between 1 and 3, with higher numbers meaning more positive experiences of ageing, and lower numbers meaning more negative experiences of ageing. The resulting measure is relational, as the respondents themselves decide what “(un)like before” means for them with respect to the dynamic of the particular life stage.

The internal consistency of the newly constructed scale was tested using Cronbach’s alpha test, with the alpha reaching 0.7292, which is acceptable. When omitting one of the three items from the calculation, the alpha decreased to 0.6287, 0.6354, and 0.6621, respectively, meaning that no more scale-purification was needed. For comparison, Lawton’s original version of the ATOA subscale showed internal consistency, with alpha estimates of 0.81 [32].

According to Cronbach’s alpha test, the POLA scale measures a one-dimensional concept. All three questions concern the same thing—positive outlook on future events related to ageing as a one form of ageing successfully and their arithmetic mean can be utilized to obtain an internally consistent scale.

### 3.1. External Validity of the Three-Item POLA Scale

In order to demonstrate the validity of our newly constructed three-item POLA scale, we analysed the relationship between the POLA scale and selected variables, both socio-demographic and self-evaluative. We presumed that if our scale truly measured positive outlook on a life development related to the ageing process as a part of morale, we would be able to find statistically significant differences between different groups of respondents. Table 2 shows the differences in mean values of the POLA scale for the categories of the three variables: data file (six categories), sex (two categories), and education (four categories). Standard statistical tests (ANOVA and *t*-test) were performed in order to test the statistical significance of the differences.

As seen in Table 2, there are statistically significant differences in mean POLA scale values among all the datasets used. Interestingly, the differences in the POLA between the average man and the average woman is quite small and not statistically significant. Despite that, the slightly more negative results for women can be accounted for by the greater average age of women in our data files due to the longer life expectancy of women (+6 years at birth) in contemporary Czech society. On the other hand, differences in the mean values of the POLA scale by educational category are significant both matter-of-factly and statistically. The higher the educational level, the better respondents were able to manage the ageing process and see it positively. The difference was greatest between primary-educated respondents and respondents who had completed secondary school without a final examination (“maturita”). At higher levels of education, the differences were smaller. It should also be noted that different topical contexts also deliver different results in the POLA with the survey on role overload and grandparenting, the second round of the Ageism survey and the survey on ageing in the biggest cities having statistically better results than the other three surveys. The Role Overload survey had many questions on various activities and roles in later life, which may have led to a revelation that “life is not over”. From a more technical or practical point of view, this survey has also the lowest average age of respondents, which represents an important intervening variable per se. In the case of Ageism 2007, we tapped for the first time in our cultural context into the issues of ageism, finding that the levels of perceived and reported age discrimination were much lower than in the later Ageism 2012. The optimism could have been slightly higher in the first survey period than in the second, when the levels of age discrimination notably rose. Unfortunately, none of these explanations works for the differences in the Quality of Life in the City 1 and the Quality of Life in the City 2 surveys. They were collected at almost the same time, and identical topics were asked and the second, more “positive” sample was slightly older. One of the underlying reasons could be the structural disparities in the living conditions and opportunities for “an active and positive” ageing experience in smaller cities outside the largest regional centers [51], which were only covered in the second survey. Here, people may have both better opportunity structures to exercise their pep and morale, as well as have, on average, more favorable characteristics, such as higher formal education, which leads to slightly better results in the POLA.

Subsequently, we used four continuous variables (which were not used to construct our three-item morale scale) to test the external validity of the three-item POLA scale. Bivariate correlation (Pearson’s r) was used to show the strength of the relations between the POLA scale and the respective variables. As a first test variable, we used the physical age of respondents (varying from 60 to 97 with a mean of 69.38). The correlation between physical age and morale as measured on the scale is negative and strong (Pearson’s r = −0.2522, *p* = 0.0000). The older the respondents, the less they seem to be eager about the ageing process. The second variable used to test the scale’s validity was respondents’ subjective age. This variable varied from 1 to 5 (mean 2.590) with higher numbers indicating respondents felt subjectively younger and lower numbers indicating they felt subjectively older. The correlation between subjective age and the POLA scale was positive and strong (Pearson’s r = 0.3981, *p* = 0.0000). Respondents with higher morale (i.e., accepting their ageing more positively) felt subjectively younger than respondents with lower morale. Another continuous variable suitable to test external validity is subjective health evaluation. Respondents were asked whether they felt subjectively healthy or unhealthy (coded as 1 and 10 respectively) or fell into any category (2–9) between these two points (mean 5.584). The correlation between subjective health evaluation and morale scale was negative and strong (Pearson’s r = −0.4239, *p* = 0.0000). Respondents with higher morale felt subjectively healthier, or expressed the other way, respondents who felt subjectively healthier had higher morale. The last variable we used to test our three-item POLA scale was loneliness. Respondents were asked to evaluate their subjective feelings of loneliness, a variable that varies from 1 (meaning “I never feel lonely”) to 10 (meaning “I always feel lonely”) with a mean of 4.281. The correlation between loneliness and morale was negative and strong (Pearson’s r = −0.4404, *p* = 0.0000). Respondents with higher morale felt less lonely than respondents with lower morale, as measured by the POLA scale.

According to the external validity test, our newly constructed three-item scale truly measures the relevant aspects of the morale concept, i.e., answers to the question of whether (or how much) the respondent views his or her ageing process positively. We have shown that the morale scale decreased with the level of education—more educated people were more positive even when growing older. Correlation tests indicated that older respondents’ morale decreased in accordance with their aging (both physically and subjectively). Morale also decreased with the subjective worsening of respondents’ health and with the deepening of their feelings of loneliness. All of the aforementioned results are coherent with the expectations we had about the POLA scale, based on the previous studies presented in the first section of this article.

### 3.2. Selected Determinants of the POLA Scale

Having showed that our three-item POLA scale can be aptly used to analyse issues related to the ageing process, in the third step of our analysis, we estimated a set of regression models. The main aim of this part of the analysis was to find the determinants which influence the POLA scale. Because one of the control variables was loneliness, which was not present in the Ageism 2007 or Ageism 2012 data files, these two files were omitted from this part of the analysis. The results are summarized in Table 3.

We used our three-item positive outlook on later life and ageing (POLA) scale as a dependent variable, and adopted the linear regression model, because the scale is continuous (varying from 1 to 3). Due to the combination of data from four data files, the multi-level approach was considered and rejected for two reasons. Firstly, the number of cases on level two (surveys, data files) was too small (four). Secondly, the intraclass correlation coefficient ICC was insufficiently low (0.0026). Therefore, we used single-level linear regression (OLS model) with survey dummy variables.

In Model M1, we used only objective information about respondents—sex, education, and age. In Model M2, subjective information, such as subjective age, subjective health, and subjective loneliness, were added. In the final model M3, the interactions between sex and age, and sex and loneliness were added, because we expected the respective variables for men and women to have different influences.

The following results and interpretations are based on model M3 and are consistent with the findings presented in the previous section. With a rising level of education, the POLA scale also rises. Educated people are more positive and accept their ageing better than people with less education. As shown by correlation coefficients, even in the regression model, the self-evaluation of respondents’ health is strongly connected with POLA scale values. People without health issues also have higher morale than their counterparts with multiple health issues. Social capital and dense social networks of high subjective quality help people in older age to accept their ageing more positively. In both the correlation analysis and the regression models, the results are very coherent—people who feel lonelier have lower morale; and people without feelings of loneliness have higher morale as measured by the POLA scale.

Importantly, the impacts of age and loneliness are gender-specific. As they grow older, men view their ageing process less positively than women, and their morale decreases. A sixty-year-old man has a morale value of 1.808 (a woman of the same age has a morale value of 1.774). Man celebrating his 90th birthday, has morale value of only 1.715 (a woman of the same age still has a morale value of 1.773). This result may be caused by, for example, lower life-expectancy for men connected with a higher probability of their health deteriorating in older age [52], as negative attitudes to ageing are also confirmed to be related to higher mortality [28,53,54].

On average, men feel less lonely (a mean of 3.939) than women (a mean of 4.531). However, when becoming lonelier, men’s morale slightly decreases (morale value of 1.991 for a man who never feels lonely, and a morale value of 1.963 for a man who always feels lonely), while women’s outlook on ageing and prospective changes in later life remains stable, regardless of their subjective social isolation. 

## 4. Discussion

In this article, we have formulated an innovative way to measure morale among older respondents. Instead of the 22-item PGCM scale or even the five-item ATOA, which is time-intensive and expensive when used in empirical surveys, we have suggested a three-item POLA scale. Jung and Siedlecki [49] have also previously tried to scale down the number of items in the ATOA, proposing that the item on usefulness should be cautiously examined. In their German sample of two age groups (40–60 and 61–74 years), this item shared less variance in common with the other items. In the POLA scale, on the other hand, the usefulness has proven a good fit with the rest of the items, as omitting would decrease the Cronbach’s alpha considerably. Moreover, the feeling of usefulness is an important part of overall later life wellbeing [55], and as Grunenwald and her colleagues have repeatedly shown, low feelings of usefulness lead to a greater hazard of mortality [56]. We would therefore strongly advocate for inclusion of this item in future studies.

The scale introduced here consists of three questions, in which the respondents are asked to compare their own perception of past and present experiences and sentiments in the following categories: pep for life, feelings of usefulness, and happiness. Using Cronbach’s alpha, we have demonstrated the internal consistency of the scale. Using mean comparisons (with statistical tests such as the *t*-test and ANOVA) and the correlation coefficients, we have shown that the scale works according to our expectations. More educated, younger, with lower subjective age, healthier, and less lonely people have higher morale than their less-educated, (subjectively) older, less healthy, and more lonely counterparts. In the third part of our analysis, we had sought to find the determinants of POLA-related morale. Since we propose the POLA scale as a new approach to measure morale and positive outlook on ageing, we would need further studies conducted in other cultural contexts and language variations to discuss the results comparatively. However, the ATOA and other awareness-of-ageing-processes studies, if we would use them as a proxy to put our results into the broader context, bring very similar results. Konradt, Siebert and Wahl [25] have found ATOA to be substantially correlated with sociodemographic and health variables in their study of midlife and later life cohorts in Germany. As already mentioned earlier, the optimism in ageing perception has been linked to health, and even mortality, in several longitudinal studies [21,24,28,53,54,57], which supports our finding that subjective health is an important covariate.

Our results slightly differ with regard to the role of gender; unlike in other studies, we have found no substantial gender differences. This is, however, not unusual, as similar, culturally specific results have been found, for example, in the loneliness measures. These typically show variation by gender [58,59,60] but have been found gender invariant by a study conducted with older Czech adults [60]. Loneliness has been a considerably suitable discriminating covariate for the POLA scale, as the lack of meaningful social contacts was associated with a less positive outlook on ageing, proving that loneliness is one of the most pronounced negative experiences associated with ageing. The overall good fit of all the concepts followed here supports our argument that the POLA scale may be a very useful tool for exploring the notion of ageing processes awareness and self-perception, densely filled with meanings and experiences of (not only) older adults and well discriminating the subtle nuances of ageing as a successful, active, and productive enterprise.

Our study has, of course, some limitations which should be addressed in future studies. The first limitation is that the Czech data files were collected over a relatively short period. Future analyses should be performed with international datasets covering more countries and longer periods. More control variables could be used to test the external validity of our morale scale, such as the size of the town in which the respondents lived, objective measurements of their physical and mental health, their economic situation, and family size, to name a few. Most of these were not available in our datasets or were available only in some of the surveys we used. Further studies would need to address the age-period-cohort effect [61] and try to disentangle the role the process of ageing as such from the underlining cohort and generational impacts, including the role of the more general societal milieu for different age cohorts at different points in time and life-course positions, all of which may have an influence over the outlook on (or the perception of) ageing as a process happening “right now” in comparison to “how it was before” or “how it was expected or planned”. In our dataset, we are not able to quantify those the differences in morale and the pace and direction of change that may occur for a 70- and a 90-year-old. As the age awareness concepts are multi-layered and complex, in our approach here, the character of our data forced us to stick with age differences, but we are aware that as a result we may not be able to see a potentially important part of the whole picture. Hopefully, future use of POLA, preferably in longitudinal studies, will be able to overcome this limitation. Open for further study is also the developmental dynamics of attitudes and experiences building up the resulting morale (cf. [25]). Last, but not least, the correlation between our POLA scale and the original PGCM and ATOA scales should be calculated using datasets in which all necessary variables are available. This would, among others, also prove or dismiss our argument for using three answer options instead of the dual “yes/no” options used in ATOA.

## 5. Conclusions

In the fields of gerontology, socio-gerontology, the sociology of age and ageing, ageing studies, psychology, and related disciplines, “commonly used awareness of aging processes constructs referring to the ongoing experience of the aging process encompass concepts such as subjective age, attitudes toward one’s own aging, self-perceptions of aging, and awareness of age-related change. Awareness of aging processes also incorporates elements that are more pre-conscious in nature, such as age stereotypes and culturally held notions about the aging process” [26]. The interest within these disciplines in such concepts is driven by the ageing of the population with higher prominence of ageing-related topics on the one hand, and by the increasing importance of the quest for improved life quality and social inclusion on the other. Notwithstanding the abovementioned limitations, we are convinced that our three-item POLA scale can be used without concern in every survey, where economy or other limitations prevent the use of the full PGCM scale. As shown in previous studies, especially when interviewing older respondents, the length of the interview can influence the quality of the data obtained (cf. [62]). Finding alternatives to the 22-item scale with a three-item scale can thus be very helpful and has the potential to produce better results in older respondents by decreasing the burden placed on them by long interviews and by improving their rapport and cooperation with the interviewer [63].

As a result, the abovementioned concepts will prove useful if they find ways into broadly interdisciplinary research on ageing, as well as outside academia. The tools for measuring positive outlook on ageing, openness to changes and the willingness to embrace them may provide a hint regarding powerful coping strategies which are key for achieving well-being [64]. Our proposed measure for positive outlook on ageing and later life—the POLA scale—may serve this purpose, as it seems to be discriminating well the “winners” of the ageing processes; it may be used to look for other important aspects which can then be treated by appropriate interventions, as in the case of loneliness. At the same time, the POLA scale is easy to disseminate, and works well in various thematical contexts. Based on the limited but promising analysis we have provided to support our argument on the functionality and usability of the newly proposed scale, we can also say that modifying and supporting a positive outlook on ageing and later life in older adults may have the potential to impact a broad range of health and successful ageing-related outcomes.

## Figures and Tables

**Table 1:** The basic characteristics of the data used.

Data Source	Number of Respondents	Three-Item POLA Scale (Mean)	Age (Mean)	Female (%)
Quality of Life in the City 1	1001	1.789 (0.464)	69.585 (6.961)	58.14
Quality of Life in the City 2	921	1.812 (0.489)	70.716 (8.085)	59.72
Ageism 2007	578	1.810 (0.418)	68.789 (6.339)	61.07
Ageism 2012	419	1.707 (0.454)	67.759 (5.808)	48.21
RURAL	1239	1.740 (0.454)	70.425 (7.555)	56.09
Role Overload	360	1.828 (0.415)	64.681 (3.335)	53.06
Total	4518	1.778 (0.457)	69.383 (7.179)	56.95

Note: standard deviations in parentheses. POLA = positive outlook on later life and ageing scale.

**Table 2:** The difference in the mean POLA scale according to the survey, sex, and education.

Variable	Category	Mean	SD	Significance of Statistical Test
**Data file**	Quality of Life in the City 1	1.789	0.464	ANOVA F = 6.13, *p* = 0.0000
	Quality of Life in the City 2	1.812	0.489	
	Ageism 2007	1.810	0.425	
	Ageism 2012	1.707	0.454	
	RURAL	1.740	0.454	
	Role Overload	1.828	0.415	
**Sex**	Male	1.784	0.443	*t*-test t = 0.2220, *p* = 0.8243
	Female	1.778	0.470	
**Education**	Primary	1.657	0.463	ANOVA F = 38.02, *p* = 0.0000
	Vocational	1.784	0.448	
	Secondary	1.839	0.458	
	Tertiary	1.872	0.438	

Note: SD = Standard deviation.

**Table 3:** Determinants of the POLA scale (OLS regression, significance in parentheses).

		M1		M2		M3	
Variable	Category	Coeff.		Coeff.		Coeff.	
Sex	Male	Ref.		Ref.		Ref.	
	Female	0.015	(0.327)	0.042	(0.004)	−0.135	(0.320)
Education	Primary	Ref.		Ref.		Ref.	
	Vocational	0.076	(0.000)	0.037	(0.055)	0.039	(0.047)
	Secondary	0.091	(0.000)	0.038	(0.073)	0.040	(0.063)
	Tertiary	0.128	(0.000)	0.050	(0.074)	0.052	(0.064)
Age		−0.006	(0.000)	−0.001	(0.231)	−0.003	(0.051)
Subjective age				0.091	(0.000)	0.090	(0.000)
Subjective health				−0.048	(0.000)	−0.048	(0.000)
Subjective loneliness				−0.046	(0.000)	−0.040	(0.000)
Interaction between Sex and Age	Male					Ref.	
	Female					0.003	(0.120)
Interaction between Sex and Loneliness	Male					Ref.	
	Female					−0.009	(0.100)
Survey dummies		…		…		…	
Constant		1.651	(0.000)	2.024	(0.000)	2.129	(0.000)
N		3027		3027		3027	
R^2^		0.1929		0.3176		0.3208	

Note: The analyses were controlled for the survey using dummy variables, but the appropriate coefficients are not presented in this table for clarity and simplicity.
